# Enzalutamide versus abiraterone as a first-line endocrine therapy for castration-resistant prostate cancer (ENABLE study for PCa): a study protocol for a multicenter randomized phase III trial

**DOI:** 10.1186/s12885-017-3661-2

**Published:** 2017-10-10

**Authors:** Kouji Izumi, Atsushi Mizokami, Mikio Namiki, Shogo Inoue, Nobumichi Tanaka, Yuko Yoshio, Kei Ishibashi, Manabu Kamiyama, Noriyasu Kawai, Hideki Enokida, Takashi Shima, Shizuko Takahara

**Affiliations:** 1 Department of Integrative Cancer Therapy and Urology, Kanazawa University Graduate School of Medical Science, 13-1 Takara-machi, Kanazawa, Ishikawa 920-8641 Japan; 2 Department of Urology, Institute of Biomedical and Health Science, Hiroshima University, Hiroshima, Japan; 3 Department of Urology, Nara Medical University, Nara, Japan; 4 Nephro-Urologic Surgery and Andrology, Division of Reparative and Regenerative Medicine, Institute of Medical Life Science, Mie University Graduate School of Medicine, Tsu, Japan; 5 Department of Urology, Fukushima Medical University, Fukushima, Japan; 6 Department of Urology, University of Yamanashi, Chuo, Japan; 7 Department of Nephro-urology, Nagoya City University Graduate School of Medical Sciences, Nagoya, Japan; 8 Department of Urology, Graduate School of Medical and Dental Sciences, Kagoshima University, Kagoshima, Japan; 9 Department of Urology, Toyama Prefectural Central Hospital, Toyama, Japan; 10 Innovative Clinical Research Center, Kanazawa University, Kanazawa, Japan

**Keywords:** Androgen-deprivation therapy, Hormone therapy, Endocrine therapy, Castration-resistant prostate cancer, Enzalutamide, Abiraterone, Randomized controlled trial

## Abstract

**Background:**

Both enzalutamide and abiraterone have demonstrated improved radiographic progression-free and overall survival for castration-resistant prostate cancer (CRPC) compared with placebo controls before docetaxel treatment in phase III studies. These oral agents target androgen and androgen receptor signaling and are thought to be less toxic than chemotherapy. Cross-resistance to these agents was recently reported because of their similar mechanism of action, and it is important to assess which agent is more effective to use initially for CRPC.

**Methods/design:**

The present study is a phase III, investigator-initiated, multicenter, head-to-head, randomized controlled trial investigating enzalutamide vs. abiraterone as a first-line treatment for CRPC patients. Patients will be randomly assigned to an enzalutamide or an abiraterone treatment group. The primary endpoint is the time to prostate-specific antigen progression. The target sample size is set at 100 patients per group (total, 200 patients). The study duration is 5 years, and the duration for recruitment is 2 years and 6 months.

**Discussion:**

Thus far, there have been no prospective head-to-head studies comparing enzalutamide and abiraterone. This ENABLE study will clarify which agent should be prioritized for CRPC patients and enable clinicians to decide the appropriate treatment before chemotherapy.

**Trial registration:**

University hospital Medical Information Network (UMIN) Center identifier UMIN000015529. Registrated 11/1/2014.

## Background

Prostate cancer is the most common malignancy and the second leading cause of death because of cancer in males in the United States [1]. Moreover, the number of prostate cancer patients in Japan has been increasing continuously [2]. Because androgen and androgen receptor signaling promotes prostate cancer progression, the standard treatment for patients with advanced prostate cancer employs androgen-deprivation therapy (ADT) [3, 4]. However, prostate cancer often progresses to castration-resistant prostate cancer (CRPC), a status that has acquired resistance to ADT after several years of treatment [5]. Both enzalutamide and abiraterone have demonstrated improved radiographic progression-free survival (rPFS) and overall survival (OS) compared with that with placebo controls before docetaxel treatment [6, 7]. These oral agents target androgen and androgen receptor signaling and are thought to be less toxic than chemotherapy (e.g., docetaxel and cabazitaxel). A cross-resistance to these agents was recently reported because of a similar anti-tumor mechanism, and it is important to determine which agent is more effective to use initially for CRPC patients [8, 9]. The benefit of enzalutamide was shown with respect to the time taken for prostate-specific antigen (PSA) progression (hazard ratio, 0.17), and a rate of decline of at least 50% in PSA (78% vs. 3%, *P* < 0.001) [6]. The median time to PSA progression (TTPP) was 11.1 and 5.6 months in the abiraterone and control group, respectively, with a 51% reduction in risk (hazard ratio, 0.49, *P* < 0.001) [10]. In this phase III multicenter randomized controlled trial (RCT), TTPP is set as a primary endpoint, and a head-to-head comparison between enzalutamide and abiraterone as a first-line endocrine therapy for CRPC is performed.

## Methods/design

### Aim of the study

To evaluate the efficacy of enzalutamide vs. abiraterone in the setting of a first-line treatment for CRPC patients.

### Study design

**Fig. 1:**
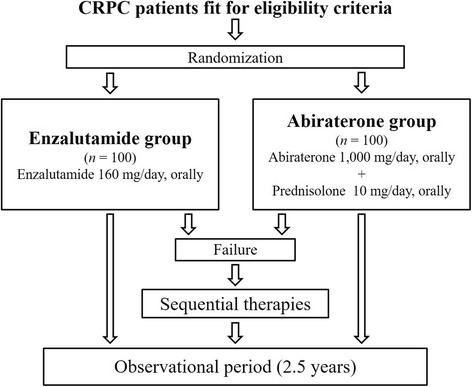
UMIN000015529

The present study is a phase III, investigator-initiated, multicenter, RCT involving a head-to-head comparison of enzalutamide vs. abiraterone for CRPC patients before chemotherapy. Patients will be randomly assigned to an enzalutamide or abiraterone treatment group as shown in Fig. 1.

### Additional measures

A validated health-related-quality-of-life questionnaire, FACT-G ver4, which has been translated into Japanese, will be administered before treatment, after the first month, and every three months after the beginning of treatment to comprehensively evaluate the various aspects of physical and psychosocial well-being.

### Eligibility criteria: Inclusion criteria

Patients must:

1. Have pathologically or cytologically confirmed CRPC, defined as total testosterone levels <50 ng/dL and two consecutive PSA elevations with a week interval, where PSA used for judgment is at least 2 ng/mL higher than nadir.
2. Have had no previous cytotoxic intravenous systemic chemotherapy.
3. Are ≥20 years when providing written informed consent.
4. Have a performance status (PS) of 0–2 according to the Eastern Cooperative Oncology Group.
5. Have appropriate hepatic and renal functionality as demonstrated in laboratory tests within four weeks prior to registration: total bilirubin level ≤ 1.5 × upper limit of normal (ULN); aspartate transaminase ≤2.5 × ULN (≤ 5.0 × ULN in patients with liver metastasis); alanine transaminase ≤2.5 × ULN (≤ 5.0 × ULN in patients with liver metastasis); and serum creatinine ≤2.0 × ULN. Neither ascites nor hepatic encephalopathy are present.
6. Have a life expectancy > three months.

### Eligibility criteria: Exclusion criteria

Patients are ineligible if they:

1. Have an allergy to enzalutamide, abiraterone, or prednisolone.
2. Have a desire to have children.
3. Are considered by a principal or clinical investigator to be inappropriate for participation in the present study for any other reason.

### Informed consent: Ethics approval

This study is conducted in accordance with the Declaration of Helsinki 1975, as revised in 2013. All treatments and examinations for prostate cancer are undertaken following written informed consent before registrations. The ENABLE study for prostate cancer (ENABLE study) received approval from the institutional ethics committees of the participating institutions.

### Methods of recruitment and random allocation

Recruitment began in November 2014 and is planned for completion by April 2017. Eligible patients are randomly assigned to one of two treatment groups through the data center at the Innovative Clinical Research Center, Kanazawa University (iCREK). Randomization is centrally performed by Waritsukekun (Mebix, Tokyo, Japan) using a minimization method to obtain adequate between-group balance for age category (<70 or ≥70), PS (0–1/2), status of metastasis (none, bone alone, or other than bone), and participating institution.

### Administration of enzalutamide and abiraterone

Enzalutamide at a dose of 160 mg/d (four 40 mg tablets once per day), is orally administered to patients who are assigned to the enzalutamide group. Abiraterone at a dose of 1000 mg/d (four tablets of 250 mg once per day), and 5 mg prednisolone twice per day, are orally administered to patients who are assigned to the abiraterone group. If a principal or clinical investigator considers the basic doses inappropriate for any reason, reduction of the doses is permitted. A history of any other treatments for which efficacy has not been shown in RCT to date is permitted, with the exception of cytotoxic intravenous chemotherapies. The administration of enzalutamide or abiraterone + prednisolone is terminated when: 1) PSA progression is confirmed; 2) the patient dies; or 3) severe adverse events occur. Luteinizing hormone-releasing hormone agonist (or antagonist) is continued throughout the study. Zoledronic acid and denosumab are permitted for patients with bone metastasis. Any sequential treatments are permitted after the confirmation of PSA progression in both groups.

### Data collection

**Fig. 2:**
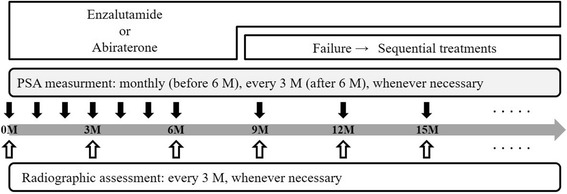
A follow-up schedule

All patients providing written informed consent to participate in the study are asked to complete a medical history. Clinical data that will be obtained in the ENABLE study include the Eastern Cooperative Oncology Group PS, physical examination findings (i.e., height, body weight, body temperature, and blood pressure), hematological test results (e.g., white blood cell, red blood cell, hemoglobin, hematocrit, and platelet counts), blood biochemical test results (e.g., total testosterone, alkaline phosphatase, bone alkaline phosphatase, total bilirubin, creatinine, liver enzymes, and electrolytes), urine test results, chest X-ray imaging, lung to pelvic computed tomography (CT) or magnetic resonance imaging (MRI), brain CT or MRI, bone scintigraphy with or without a bone scan index, electrocardiography, and the quality-of-life questionnaire, FACT-G ver4. The chest X-ray and brain CT are performed at the time of study registration. Other examinations are performed every month from the date of commencement to the sixth month, and every three months after the sixth month until the study is completed (Fig. 2). However, if a principal or clinical investigator considers these examinations to be necessary, they can be performed at any time.

### Definition of endpoints

The primary endpoint is TTPP, defined on the basis of prostate cancer working group 2 (PCWG2) criteria [5] as described briefly below. For patients in whom PSA declines at week 13, PSA progression date is defined as the date that a ≥ 25% increase and an absolute increase of ≥2 ng/mL above the nadir is documented. This increase was confirmed by a second consecutive value obtained at least three weeks later. For patients without a PSA decline at week 13, PSA progression date was defined as the date that a ≥ 25% increase and an absolute increase of ≥2 ng/mL above the baseline is documented. This is confirmed by a second consecutive value, at least three weeks later. For all patients, TTPP was defined as the time from randomization to first confirmed PSA progression.

Eight secondary endpoints are set in ENABLE study as follows:

1. OS, defined as the time from randomization to death from any cause.
2. rPFS based on the Response Evaluation Criteria in Solid Tumors (RECIST) criteria for soft-tissue lesions examined with CT or MRI and PCWG2 criteria for bone metastasis examined with bone scintigraphy.
3. Time to the commencement of cytotoxic chemotherapy (e.g., docetaxel and cabazitaxel).
4. Time to stage progression in PS.
5. Time to the commencement of opioid analgesics for cancer pain.
6. PSA response rate (≥50% decline in PSA level from baseline).
7. Safety according to the frequency and grade using Common Terminology Criteria for Adverse Events (CTCAE), Version 4.0 (http://evs.nci.nih.gov/ftp1/CTCAE/About.html).
8. Health-related quality-of-life using FACT-G ver4.

Three exploratory endpoints are set in ENABLE study:

1. The type of secondary treatment.
2. OS from the randomization of the ENABLE study as a sequential therapy, in case the secondary treatment is docetaxel.
3. TTPP, rPFS, and PSA response rate after the commencement of secondary treatment.

### Planned statistical analyses

TTPP of the treatment and control group in the studies on enzalutamide and abiraterone before chemotherapy was 11.2 vs. 2.8 months and 11.1 vs. 5.6 months, respectively [6, 7, 10]. The addition of prednisolone is required to compensate for the decrease in cortisol levels due to abiraterone. Prednisolone has been reported to have a moderate anti-tumor effect in prostate cancer patients and to extend 2 months in TTPP [11]. As prednisolone was administered to all patients in the abiraterone study, TTPP of the abiraterone treatment group may be reduced to 3.6 months. The inclusion of patients with visceral metastasis might be a potential reason for this difference between the control groups (2.8 and 3.6 months) in the two studies. If the patient backgrounds of these two studies are same, TTPP of the abiraterone treatment group before chemotherapy is 11.2 × (2.8/3.6) = 8.6 months. Using this calculated hypothetical TTPP in abraterone group, TTPP in enzalutamide group (11.2 months) is 2.6 months superior to TTPP in abiraterone group (8.6 months). We calculated the sample size from 5 years of the study duration and the difference in TTPP between the enzalutamide and abiraterone groups. The basic methods of statistical analyzes were described in a previous study [12]. At least, 91 patients in each group are required to detect a significant difference between the enzalutamide and abiraterone groups by a log-rank test with a significance level of 0.05 and a power of 80%. Furthermore, given the assumption that approximately 10% of randomized patients will not be evaluable for various reasons, the target sample size was set at 100 patients per group (total 200 patients). Intention-to-treat analyses will be performed, and survival curves will be estimated using the Kaplan–Meier method. A log-rank test will be used to test for differences in the survival curves between the two groups of patients. The hazard ratio will be estimated using the Cox proportional hazard model. Moreover, the longitudinal changes in the health-related-quality-of-life between time of diagnosis and during treatment will also be compared between the two groups. All patients will be evaluated for toxicity, and the incident proportion of grade 3 adverse events will be compared between the groups by a Fisher’s exact test. All tests will be two-sided, and a *P*-value of 0.05 will be considered statistically significant. The study will be completely analyzed two and a half years after the last patient is recruited.

### Patient enrollment and anticipated completion of enrollment

Our current expectation is that the final patient will be enrolled by April 2017, and the entire study will be completed by October 2019. Cumulative enrollment reached 40 cases as of February 2016.

## Discussion

Docetaxel has been used as a first-line treatment for CRPC after the proof of its efficacy in a randomized phase III study in 2004 [6, 7]. Although the Japanese government approved docetaxel for CRPC in 2008 and it is often used clinically, it is a cytotoxic agent and can be unsuitable for treating older patients and those with co-morbidities. There was no other treatment for which efficacy was proven by a phase III study until 2010. Cabazitaxel emerged as a second-line treatment for CRPC for the first time in 2010 [13]. Subsequently, the efficacies of four treatment lines, enzalutamide, abiraterone, sipuleucel-T, and radium-223, were demonstrated by phase III studies in consecutive publications [6, 10, 14, 15]. There has been no head-to-head study performed for these novel treatments for CRPC, which complicates the CRPC treatment decisions taken by clinicians. Previously, prostate cancer in ADT-resistant patients was referred to as hormone-refractory prostate cancer. It was reported that prostate cancer cells take advantage of the low levels of androgens after ADT, and androgens are even synthesized in the prostate cancer cells. Therefore, the term “CRPC” is currently used following confirmation of low total testosterone levels, typically defined as <50 ng/dL [5]. Abiraterone inhibits CYP17A1 (both 17a–hydroxylase and 17,20-lyase) in androgen biosynthesis, whereas enzalutamide binds to the androgen receptor with a greater relative affinity than conventional anti-androgen agents, reduces the efficiency of its nuclear translocation, and impairs both DNA binding to androgen response elements and recruitment of coactivators [16, 17]. Therefore, it was considered to be of great value to compare these two orally administered hormonal treatments with low toxicity.

TTPP was set as a primary endpoint in the ENABLE study, which differed from previous studies that used OS and rPFS as the primary endpoints [6, 7, 10]. The ENABLE study was planned to investigate patients before chemotherapy, and the OS may substantially depend on subsequent treatments. Moreover, conventional hormonal manipulations (e.g., ethinylestradiol, estramustine phosphate, and dexamethasone) can also have an anti-tumor effect, and may extend the OS if used after the ENABLE study [18–20]. Although it may be universal for studies of cancer to use rPFS with RECIST, it may not be applied to an advanced prostate cancer study due to its extremely high frequency of bone metastasis [21]. If rPFS is used as a primary endpoint, the quantitation of bone metastasis is necessary to correctly assess the disease status [22]. Interestingly, 43% of cases of cancer progression could be detected by PSA but not radiographic progression. Nevertheless, only 13% of cases of cancer progression could be detected by radiographic progression alone [6]. Although PSA has potential limitations as reported previously, the PSA assay is extremely easy to perform, is relatively inexpensive, and less invasive because PSA consists of secreted proteins present in the blood [23–25].

Recently, although a prospective phase II study comparing enzalutamide and abiraterone (NCT02125357) showed no difference in time to PSA progression [26], this is a cross-over study of abiraterone vs enzalutamide and still ongoing. There have been no prospective head-to-head phase III studies comparing enzalutamide and abiraterone conducted to date. The ENABLE study is the first study of its kind, will clarify which agent should have priority for CRPC patients, and will enable clinicians to decide the most appropriate treatment before chemotherapy.

## Abbreviations

ADT: Androgen-deprivation therapy
CRPC: Castration-resistant prostate cancer
CT: Computed tomography
CTCAE: Common Terminology Criteria for Adverse Events
MRI: magnetic resonance imaging
OS: overall survival
PCWG2: prostate cancer working group 2
PS: Performance status
RCT: Randomized controlled trial
RECIST: Response Evaluation Criteria in Solid Tumors
rPFS: Radiographic progression-free survival
TTPP: Time to PSA progression
ULN: upper limit of normal
